# Mitogenomes provide new insights of evolutionary history of Boreheptagyiini and Diamesini (Diptera: Chironomidae: Diamesinae)

**DOI:** 10.1002/ece3.8957

**Published:** 2022-05-24

**Authors:** Xiao‐Long Lin, Zheng Liu, Li‐Ping Yan, Xin Duan, Wen‐Jun Bu, Xin‐Hua Wang, Chen‐Guang Zheng

**Affiliations:** ^1^ 12538 College of Life Sciences Nankai University Tianjin China; ^2^ 74518 Geological Museum of China Beijing China; ^3^ 12380 School of Ecology and Nature Conservation Beijing Forestry University Beijing China; ^4^ 91633 Department of Plant Protection College of Horticulture and Landscape Tianjin Agricultural University Tianjin China

**Keywords:** Diamesinae, mitogenome, phylogeny

## Abstract

Mitogenomes have been widely used for phylogenetic reconstruction of various Dipteran groups, but specifically for chironomid, they have not been carried out to resolve the relationships. Diamesinae (Diptera: Chironomidae) are important bioindicators for freshwater ecosystem monitoring, but its evolutionary history remains uncertain for lack of information. Here, coupled with one previously published and 30 new mitogenomes of Diamesinae, we carried out comparative mitogenomic analysis and phylogenetic analysis. Mitogenomes of Diamesinae were conserved in structure, and all genes arranged in the same order as the ancestral insect mitogenome. All protein‐coding genes in Diamesinae were under stronger purifying selection than those of other nonbiting midge species, which may exhibit signs of adaptation to life at cold living conditions. Phylogenetic analyses strongly supported the monophyly of Diamesinae, with Boreheptagyiini deeply nested within Diamesini. In addition, phylogenetic relationship of selected six genera was resolved, except *Sympotthastia* remained unstable. Our study revealed that the mitogenomes of Diamesinae are highly conserved, and they are practically useful for phylogenetic inference.

## Graphical abstract

Here, coupled with one previously published and 30 new mitogenomes of Diamesinae, we carried out comparative mitogenomic analysis and phylogenetic analysis. Mitogenomes of Diamesinae were conserved in structure, and all genes arranged in the same order as the ancestral insect mitogenome. Phylogenetic analyses strongly supported the monophyly of Diamesinae, with Boreheptagyiini deeply nested within Diamesini.

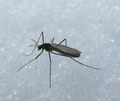

## 1 INTRODUCTION

**FIGURE 1:**
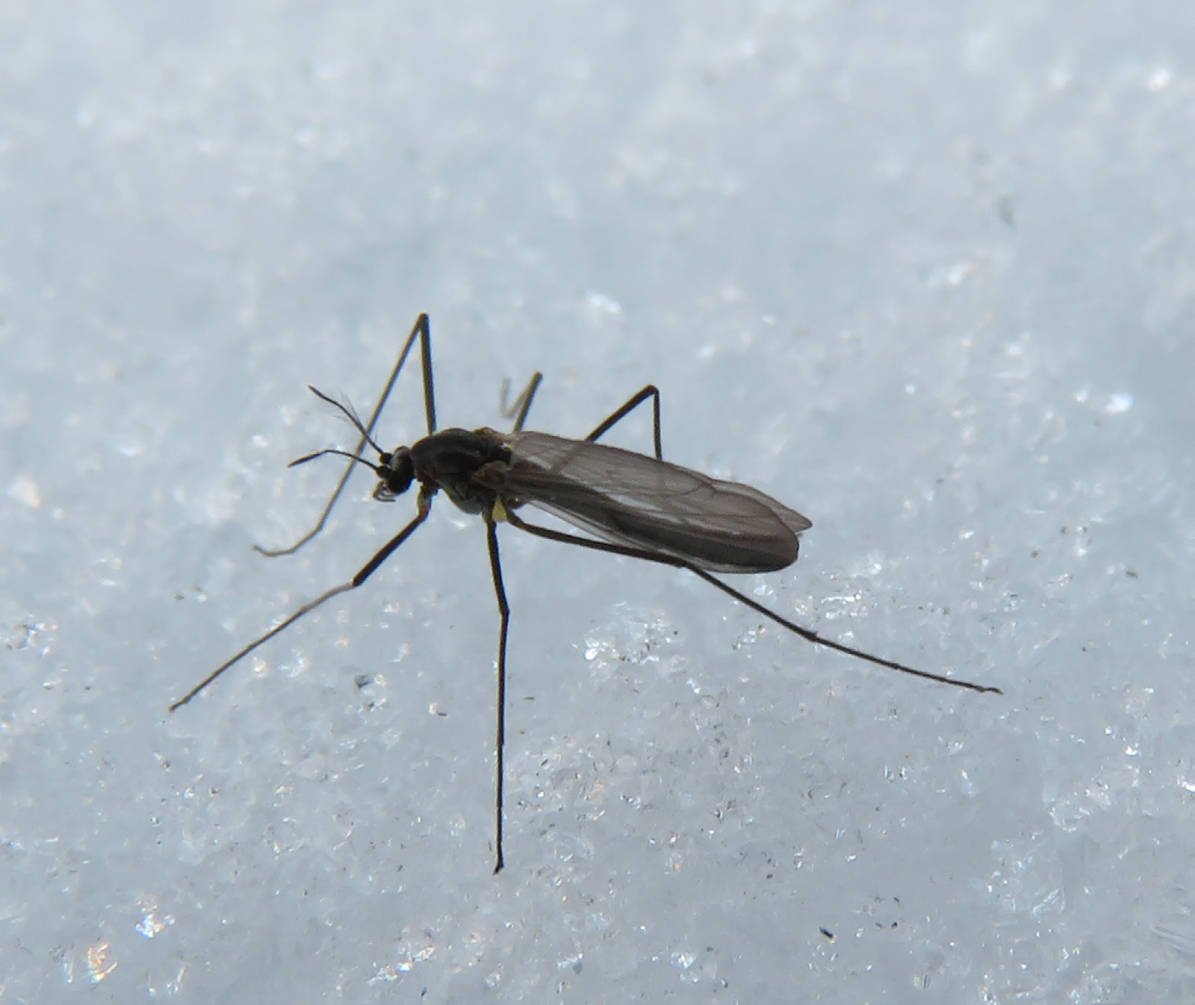
An adult male of *Diamesa loeffleri* Reiss, 1968 on the ice in Qinghai, China. Photo: Qing‐Bo Huo

Dipteran family Chironomidae have the most abundant species richness among freshwater macroinvertebrates, including more than 6300 species worldwide, even in Antarctica (Kelley et al., 2014; Kim et al., 2016). Since their great species diversity and ability to inhabit different types of water body, chironomid larvae are key bioindicators for freshwater ecosystem monitoring. Several phylogenetic studies have been conducted based on morphological characters or combining genetic markers to reconstruct the evolutionary history of Chironomidae (Brundin, 1966; Cranston et al., 2012; Cranston & Krosch, 2015; Ekrem, 2003; Krosch & Cranston, 2013; Lin et al., 2018; Qi et al., 2019; Sæther, 1977, 2000; Serra‐Tosio, 1973; Silva et al., 2015), but few has attempted to use mitogenomes. Diamesinae (Figure 1) is a relatively small subfamily within Chironomidae, containing over 100 species of six tribes: Boreheptagyiini, Diamesini, Harrisoniini, Heptagyiini, Lobodiamesini, and Protanypini (Ashe & O'Connor, 2009; Brundin, 1966; Sæther, 2000). At present, Boreheptagyiini includes three genera (*Boreoheptagyia* Brundin, *Palatovia* Makarchenko & Semenchenko, and *Shilovia* Makarchenko) distributed in Holarctic and Oriental regions (Makarchenko et al., 2017). Diamesini contains 11 genera: *Arctodiamesa* Makarchenko, *Diamesa* Meigen, *Kaluginia* Makarchenko, *Lappodiamesa* Serra‐Tosio, *Pagastia* Oliver, *Potthastia* Kieffer, *Pseudodiamesa* Goetghebuer, *Pseudokiefferiella* Zavrel, *Sasayusurika* Makarchenko, *Sympotthastia* Pagast, and *Syndiamesa* Kieffer (Ashe & O'Connor, 2009) distributed in Afrotropical, Holarctic, and Oriental regions. Phylogenetic relationships within Diamesinae are still controversial despite more than 50 years of research. Traditionally, the phylogenetic relationships of Diamesinae were inferred by morphological characters (Brundin, 1966; Sæther, 1977, 2000). Until last decade, the phylogenetic relationship of very limited sets of Diamesinae subgroups has been explored based on a few molecular loci (Cranston et al., 2012; Lencioni et al., 2021; Montagna et al., 2016). However, Boreheptagyiini and Protanypini were missing, and Diamesini taxa were undersampled in their study. Therefore, the phylogenetic relationship within Diamesini and Boreheptagyiini was recovered by morphological characters is misleading.

In general, mitogenomes of most insects is a double‐strand circular DNA molecule ranging from 14 kb to 20 kb in size, encoding 37 genes (13 protein‐coding genes, two ribosomal RNA genes, and 22 transfer RNA genes) and a control region (Boore, 1999; Cameron, 2014; Wolstenholme, 1992). Since its small genome size, maternal inheritance, low sequence recombination, and fast evolutionary rates (Brown et al., 1979; Curole & Kocher, 1999), the mitogenome is considered as powerful marker for phylogenetic and evolutionary analysis in many insect groups (Condamine et al., 2018; Crampton‐Platt et al., 2015; Jacobsen et al., 2012; Tang, Zhu, et al., 2019; Yan et al., 2019). Benefiting from the advances of high‐throughput sequencing technology, an increasing number of complete mitogenomes have been sequenced among the Diptera (Kang et al., 2016; Li et al., 2020; Miao et al., 2020; Ramakodi et al., 2015; Tang, Yan, et al., 2019; Wang et al., 2021; Yan et al., 2021; Zhang et al., 2022), and have been widely used for mitochondrial structure comparison and phylogenetic analysis at different taxonomic levels (Chen et al., 2018; de Oliveira Aragão et al., 2019; Yan et al., 2019; Zhang et al., 2016; Zhang, Kang, et al., 2019). Prior to this study, rare mitogenomes of Chironomidae were available (Beckenbach, 2012; Deviatiiarov et al., 2017; Fang et al., 2022; Jiang et al., 2022; Kim et al., 2016; Kong et al., 2021; Lei et al., 2021; Park et al., 2020; Zhang, Xu, et al., 2019; Zheng et al., 2022; Zheng et al., 2021), limiting our understanding of their mitochondrial structure and phylogenetic pattern. Besides, it is still unknown whether mitogenomes can effectively resolve phylogenetic relationships at different levels within Chironomidae. To date, only one mitogenome of Diamesinae was available, representing Diamesini (Zheng et al., 2021).

In this study, we provide 30 newly sequenced (nearly) complete mitogenomes from 30 species representing Boreheptagyiini (four species of one genus) and Diamesini (26 species of five genera) using next‐generation sequencing. We analyzed the genomic structure, base composition, substitution, and evolutionary rates among Diamesinae, expanding our knowledge of its diversity of mitogenomes. Coupled with published data, we carried out phylogenetic analysis of Boreheptagyiini and Diamesini based on 31 mitogenomes.

## 2 MATERIALS AND METHODS

### 2.1 Taxon sampling and dna extraction

Field collection of 30 species were conducted in China during 2014–2020, using classical insect collection techniques such light traps, sweep traps, Malaise traps, and D‐nets. Specimens were preserved in ethanol (85% for adults, 95% for immature), and stored at dark at −20°C before morphological and molecular analyses. The total genomic DNA was extracted from thorax of adult and middle larval bodies using a Qiagen DNA Blood and Tissue Kit (Qiagen) following the manufacturer's protocol. After DNA extraction, the cleared exoskeleton of thorax was mounted in Euparal on microscopy slides together with the corresponding wings, legs, and antennae following the procedures outlined by Sæther (1969). The DNA and vouchers of the species are deposited at the college of Life Sciences, Nankai University, Tianjin, China. Specimens were identified morphologically using relevant taxonomic revisions and species descriptions (Lin, Chang, et al., 2021; Lin, Yu, et al., 2021; Makarchenko et al., 2008, 2021; Makarchenko & Wang, 2017; Moubayed‐Breil & Orsini, 2016; Oliver, 1983, 1989; Reiss, 1968; Sun et al., 2019), belonging to two tribes of Diamesinae.

Thirty mitogenomes were newly sequenced in this study, representing four species of Boreheptagyiini (four *Boreoheptagyia* species) and 26 species of Diamesini (15 *Diamesa* species, four *Pagastia* species, four *Potthastia* species, two *Pseudodiamesa* species and one *Sympotthastia* species). Since mitogenomes of another four tribes were not available for current molecular study, we could not reconstruct the phylogeny of the whole subfamily Diamesinae. Therefore, by integrating one public *Potthastia* species (GenBank accession: MW373523), a total of 31 species of Boreheptagyiini and Diamesini were selected as in‐groups. In addition, we selected one Prodiamesinae species (*Prodiamesa olivacea* [Meigen, 1818], GenBank accession: MW373525) and one Orthocladiinae species (*Propsilocerus akamusi* [Tokunaga, 1938], GenBank accession: MW846253) as outgroups for phylogenetic analyses. Detailed information could be found in Table 1. Each sample ID in Table 1 represents the voucher unique identifier.

**TABLE 1:** Taxonomic information, sampling metadata, GenBank accession numbers, and references of mitochondrial genomes used in the study

Sample ID	Subfamily	Species	Sampling metadata	Life stage	Accession no	Reference
XL3275	Prodiamesinae	*Prodiamesa olivacea*	Jiuzhaigou Valley Scenic and Historic Interest Area, Sichuan, China, 33.1928°N, 103.8942°E, 12‐Jul‐2019, leg. X.‐Y. Ge	Larva	MW373525	Lin et al. (2022)
XL3436	Orthocladiinae	*Propsilocerus akamusi*	Yuqiao Reservoir, Jizhou, Tianjin, China, 40.0197°N, 117.6389°E, 21‐Nov‐2019, leg. H.‐J. Yu	Adult male	MW846253	Lin et al. (2022)
XL1177	Diamesinae	*Boreoheptagyia alulasetosa*	Cangshan Mountain, Dali, Yunnan, China, 25.6475°N, 100.1426°E, 20‐May‐2018, leg. X.‐L. Lin	Adult male	MZ043574	This study
ZJ837	Diamesinae	*Boreoheptagyia brevitarsis*	Lingdi, Wenzhou, Zhejiang, China, 28.3276°N, 120.8774°E, 5‐May‐2019, leg. X.‐L. Lin	Adult male	MZ043575	This study
LGS62	Diamesinae	*Boreoheptagyia kurobebrevis*	Leigongshan Natural Reserve, Guizhou, China, 26.3960°N, 108.2609°N, 20‐Jan‐2020, leg. H.‐J. Yu	Adult female	MZ043576	This study
XL3519	Diamesinae	*Boreoheptagyia zhengi*	Gaoligongshan National Nature Reserve, Baoshan, Yunnan, China, 25.3106°N, 98.7950°E, 22‐May‐2018, X.‐L. Lin	Adult male	OM302508	This study
XL4059	Diamesinae	*Diamesa loeffleri*	Shoule town, Haidong, Qinghai, China, 36.7707°N, 102.4887°E, 26‐Nov‐2020, leg. Q.‐B. Huo	Adult male	MZ127838	This study
CHMIT19	Diamesinae	*Diamesa qiangi*	Lulang, Xizang, China, 29.77°N, 94.74°E, 14‐Aug‐2013, leg. Q. Wang	Adult male	MZ127839	This study
XL4057	Diamesinae	*Diamesa* sp. 1XL	Shoule town, Haidong, Qinghai, China, 36.7707°N, 102.4887°E, 26‐Nov‐2020, leg. Q.‐B. Huo	Larva	MZ048035	This study
XL3288	Diamesinae	*Diamesa* sp. 2 XL	Huanglong Scenic and Historic Interest Area, Sichuan, China, 30.72538°N, 103.8331°E, 17‐Jul‐2019, leg. X.‐Y. Ge	Larva	MZ048036	This study
XL2214	Diamesinae	*Diamesa* sp. 3XL	Shangchayuzhen, Zayu, Xizang, China, 28.73868694°N, 96.76293611°E, 24‐Mar‐2016, leg. Z.‐Y. Liu	Larva	MZ048037	This study
XL1967	Diamesinae	*Diamesa* sp. 4XL	Zhongshacun, Mainling, Xizang, 29.1873°N, 93.9954°E, 16‐Jul‐2014, leg. X.‐L. Lin	Larva	MZ048038	This study
XL3464	Diamesinae	*Diamesa* sp. 5XL	Erdaobaihezhen, Antu, Jilin, China, 42.4011°N, 128.1008°E, 22‐Aug‐2019, leg. S. Qiu	Larva	MZ231027	This study
XL2212	Diamesinae	*Diamesa* sp. 6XL	Shangchayuzhen, Zayu, Xizang, China, 28.7387°N, 96.76294°E, 24‐Mar‐2016, leg. Z.‐Y. Liu	Larva	MZ158293	This study
XL1930	Diamesinae	*Diamesa* sp. 7XL	Bomi, Xizang, China, 29.8035°N, 95.8672°E, 11‐Jul‐2014, leg. X.‐L. Lin	Larva	MZ158294	This study
XL2216	Diamesinae	*Diamesa* sp. 8XL	Shangchayuzhen, Zayu, Xizang, China, 28.7387°N, 96.7629°E, 24‐Mar‐2016, leg. Z.‐Y. Liu	Larva	MZ231028	This study
XL3286	Diamesinae	*Diamesa* sp. 9XL	Huanglong Scenic and Historic Interest Area, Sichuan, China, 30.7253°N, 103.8331°E, 17‐Jul‐2019, leg. X.‐Y. Ge	Larva	MZ231029	This study
XL2133	Diamesinae	*Diamesa* sp. 10XL	Baiyanggou, Qinghai, China, 38.2283°N, 100.2674°E, 24‐Jul‐2019, leg. X.‐J. Zhu	Adult male	MZ043577	This study
XL1929	Diamesinae	*Diamesa* sp. 11XL	Bomi, Xizang, China, 29.8035°N, 95.8672°E, 11‐Jul‐2014, leg. X.‐L. Lin	Larva	MZ043578	This study
XL1907	Diamesinae	*Diamesa* sp. 12XL	Ranwu Lake, Chamdo, Xizang, China, 29.5050°N, 96.7489°E, 10‐Jul‐2014, leg. X.‐L. Lin	Larva	MZ158295	This study
XL2121	Diamesinae	*Diamesa tonsa*	Qihan, Qinghai, China, 37.1555°N, 102.0238°E, 17‐Apr‐2019, leg. X.‐J. Zhu	Adult male	MZ158292	This study
XL877	Diamesinae	*Pagastia lanceolata*	Gaoligongshan National Nature Reserve, Baoshan, Yunnan, China, 25.3106°N, 98.7950°E, 22‐May‐2018, leg. X.‐L. Lin	Adult male	OM302510	This study
XL3361	Diamesinae	*Pagastia* sp. 1XL	Sangzhuziqu, Xizang, 12‐Aug‐2019, leg. J. Jiang	Larva	OM302507	This study
XL3290	Diamesinae	*Pagastia* sp. 2XL	Huanglong Scenic and Historic Interest Area, Sichuan, China, 30.7253°N, 103.8331°E, 17‐Jul‐2019, leg. X.‐Y. Ge	Larva	OM302505	This study
XL3460	Diamesinae	*Pagastia tianmumontana*	Erdaobaihezhen, Antu, Jilin, China, 42.4011°N, 128.1008°E, 22‐Aug‐2019, leg. S. Qiu	Larva	MZ231025	This study
XL3152	Diamesinae	*Potthastia gaedii*	Fanjingshan National Nature Reserve, Tongren, Guizhou, China, 27.7392°N, 108.8212°E, 7‐Oct‐2019, leg. H.‐J. Yu	Larva	OM302504	This study
LGS11	Diamesinae	*Potthastia* sp. 1XL	Xianlvtang, Leigongshan Natural Reserve, Guizhou, China, 20‐Dec‐2019, leg. H.‐J. Yu	Adult male	OM302509	This study
XL1347	Diamesinae	*Potthastia* sp. 2XL	Juma River, Baoding, Hebei, China, 39.4280°N, 115.1701°E, 8‐May‐2018, leg. X.‐L. Lin	Adult male	MZ064641	This study
XL1561	Diamesinae	*Potthastia* sp. 3XL	Wuying River, Yichun, Heilongjiang, China, 48.0869°N, 129.2468°E, 27‐Jul‐2016, leg. C. Song	Adult male	MW373523	Zheng et al. (2021)
XL1623	Diamesinae	*Potthastia* sp. 4XL	Erdaobaihezhen, Antu, Jilin, China, 42.4567°N, 128.1442°E, 12‐Jul‐2016, leg. C. Song	Adult male	OM302503	This study
XL2282	Diamesinae	*Pseudodiamesa* sp. 1XL	Songduo Mian Steam, Xizang, China, 29.9067°N, 92.3981°E, 14‐Oct‐2016, leg. Z.‐Y. Liu	Larva	MZ064643	This study
XL3334	Diamesinae	*Pseudodiamesa* sp. 2XL	Huanglong Scenic and Historic Interest Area, Sichuan, China, 30.7253°N, 103.8331°E, 16‐Jul‐2019, leg. X.‐Y. Ge	Larva	OM302506	This study
ZJ283	Diamesinae	*Sympotthastia takatensis*	Lingdi, Wenzhou, Zhejiang, China, 28.3044°N, 120.9295°E, 7‐Apr‐2019, leg. X.‐L. Lin	Pupa	MZ231026	This study

### 2.2 Sequencing and mitogenome assembly

The whole genomes were sequenced using the Illumina NovaSeq 6000 platform with 150‐bp paired‐end reads at Novogene Co., Ltd. (Beijing, China). The raw sequencing reads were trimmed with Trimmomatic (Bolger et al., 2014), and then about two Gb of clean data were obtained for each sample. The clean data were assembled using IDBA‐UD (Peng et al., 2012) with minimum and maximum k values of 40 and 120 bp, respectively, and the similarity was set as 98%.

The cytochrome *c* oxidase I (COI) barcode sequence for each species was obtained by Sanger sequencing herein and from previous study (Lin, Yu, et al., 2021), and served as the “bait” references to acquire the best‐fit and targeted mitochondrial contigs by BLAST (Altschul et al., 1990) search in Geneious 2020.2.1 (Kearse et al., 2012). Moreover, clean reads were mapped onto the obtained mitogenome using Geneious to check the accuracy of the assembly.

### 2.3 Genome annotation, composition, and substitution rate

Genome annotation was conducted following previous study (Zheng et al., 2020). Transfer RNA (tRNA) genes and their secondary structures were identified on MITOS2 webserver (available at http://mitos2.bioinf.uni‐leipzig.de/index.py). Ribosomal RNA (rRNA) genes and protein‐coding genes (PCGs) were annotated by aligning with homologous genes of *Potthastia* sp. in Geneious. Newly sequenced mitogenomes were submitted to GenBank (accession numbers: pending). The mitogenome maps were drawn by the CG View server V 1.0 (Grant & Stothard, 2008). The base composition, codon usage, and relative synonymous codon usage (RSCU) values were calculated in MEGA X (Kumar et al., 2018). The bias of the nucleotide composition was measured by AT‐skew [(A − T)/(A + T)] and GC‐skew [(G − C)/(G + C)]. The ratio (ɷ) of nonsynonymous substitution rates (Ka) to Synonymous substitution rates (Ks) was an excellent estimator of evolutionary selection pressure. Synonymous substitution rates (Ks) and nonsynonymous substitution rates (Ka) of mitochondrial PCGs were calculated using DnaSP 6.12.03 (Rozas et al., 2017).

### 2.4 Substitution rate and phylogenetic analyses

The level of base substitution saturation for each gene and each position of the PCGs was assessed using DAMBE 5.6.14 (Xia, 2013). Substitution of each of the three codon positions are generally not saturated, except for the transition of 3rd codon positions (Figure S1). Therefore, the 3rd codon positions of PCGs were excluded for the phylogenetic analyses. Each gene was aligned using MAFFT 7.402 (Katoh & Standley, 2013) with algorithm G‐INS‐i strategy. Gap in each matrix was treated as the fifth character and was retained in this study. Alignments of individual genes were then concatenated using SequenceMatrix v1.7.8 (Vaidya et al., 2011), after which three datasets were prepared for phylogenetic analyses: PCG12 (the 1st and 2nd codon positions of the 13 PCGs), PCG12R (the 1st and 2nd codon positions of the 13 PCGs and two rRNAs), and third AA (amino acid sequences of the 13 PCGs). The best partitioning scheme and best‐fit substitution model for each partition was tested using PartitionFinder 2.0 (Lanfear et al., 2017) with the Bayesian Information Criterion (BIC). Phylogenetic analyses were conducted with Maximum likelihood (ML) reconstruction and Bayesian inference (BI). The ML analysis was performed using IQ‐TREE 1.6.10 (Nguyen et al., 2015) with the best‐fit substitution model and 1000 bootstrap replicates. BI analysis was performed using MrBayes 3.2.7a (Ronquist et al., 2012) with substitution model in Table S1. Two simultaneous Markov chain Monte Carlo (MCMC) runs of 10,000,000 generations were conducted, trees were sampled every 1000 generations, and the first 25% of trees discarded as burn‐in. Tracer 1.7 (Rambaut et al., 2018) was used to check convergence of runs.

## 3 RESULTS

### 3.1 Mitogenome features of Diamesinae

**FIGURE 2:**
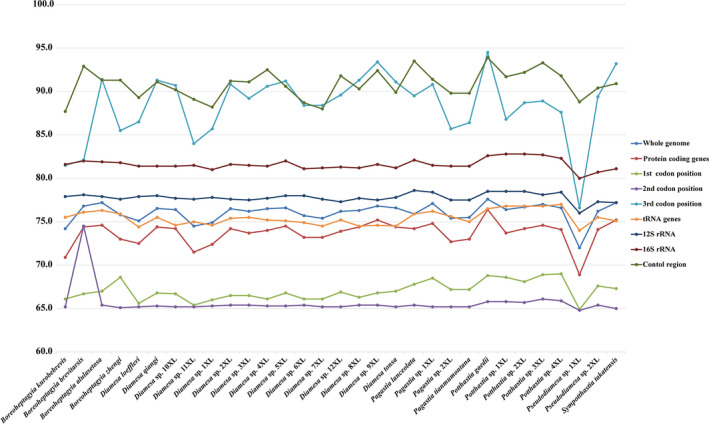
A+T content of mitochondrial genes of Diamesinae species. The X‐axis shows the species names and the *Y*‐axis shows the percentage of A+T content

**FIGURE 3:**
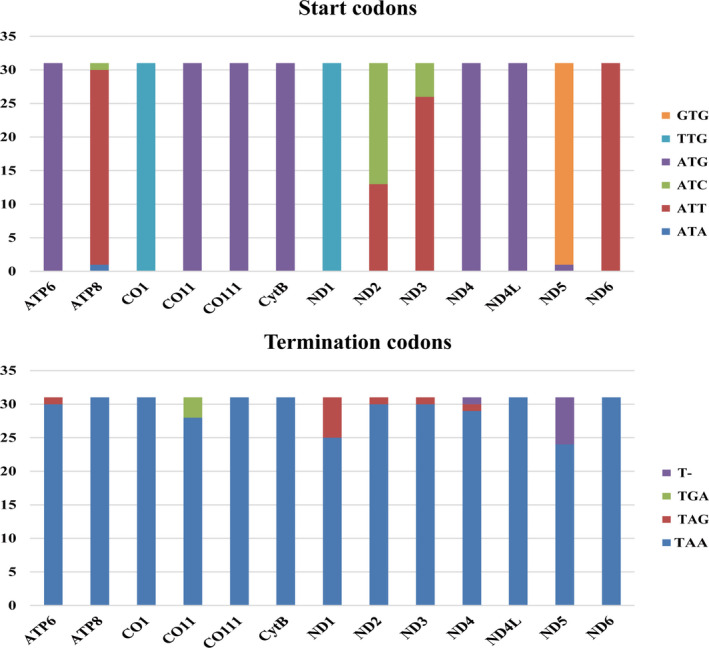
Start and termination codons of PCGs among Diamesinae species. The X‐axis shows the names of PCGs and the *Y*‐axis shows the codon numbers

The mitogenomes of 31 Diamesinae species were included in this study, 21 of which are complete, with the entire length ranging from 15,913 bp to 16,411 bp (Table S2). Each mitogenome contains 37 genes (13 PCGs, two rRNAs, and 22 tRNAs) and one control region. Nine PCGs, 14 tRNAs, and 2 rRNAs are coded on the majority strand (J‐strand), while the other genes are coded on the minority strand (N‐strand). The A + T content of the whole mitogenomes ranged from 72% in *Pseudodiamesa* sp. 1XL to 77.6% in *Potthastia gaedii* (Meigen, 1838) (Figure 2). Among the mitogenomes of Diamesinae, the control region and the 3rd codon of PCGs have the highest A + T content, while the 1st and 2nd codons of PCGs exhibit the lowest A + T content. The A + T content of rRNA genes is slightly higher than that in the whole mitogenomes, PCGs, and tRNA genes (Figure 2). In all selected species, the AT‐skew value of tRNA genes is positive while that of PCGs is negative. The GC‐skew values of rRNA genes and the 1st codon of PCGs are positive, while negative in the whole mitogenomes and the 2nd codon of PCGs (Figure S2). The start codons in most PCGs of the mitogenomes among Diamesinae are ATN (N represents one of four nucleotides, A, T, C, G), while COI and ND1 start with TTG. In addition, ND5 start with GTG in most mitogenomes of Diamesinae (Figure 3). The most prevalent termination codon used in mitogenomes of Diamesinae is TAA, with a small number of PCGs terminate with TAG, TGA, and T‐ (Figure 3). The total codon numbers, except the termination codons in mitogenomes of Diamesinae range from 3565 to 3735. Leu2, Phe, and Ile are the three most frequently used codon families, each with a number of more than 300. The least frequently used codon family is Cys, with a number less than 50 (Figure S3).

**FIGURE 4:**
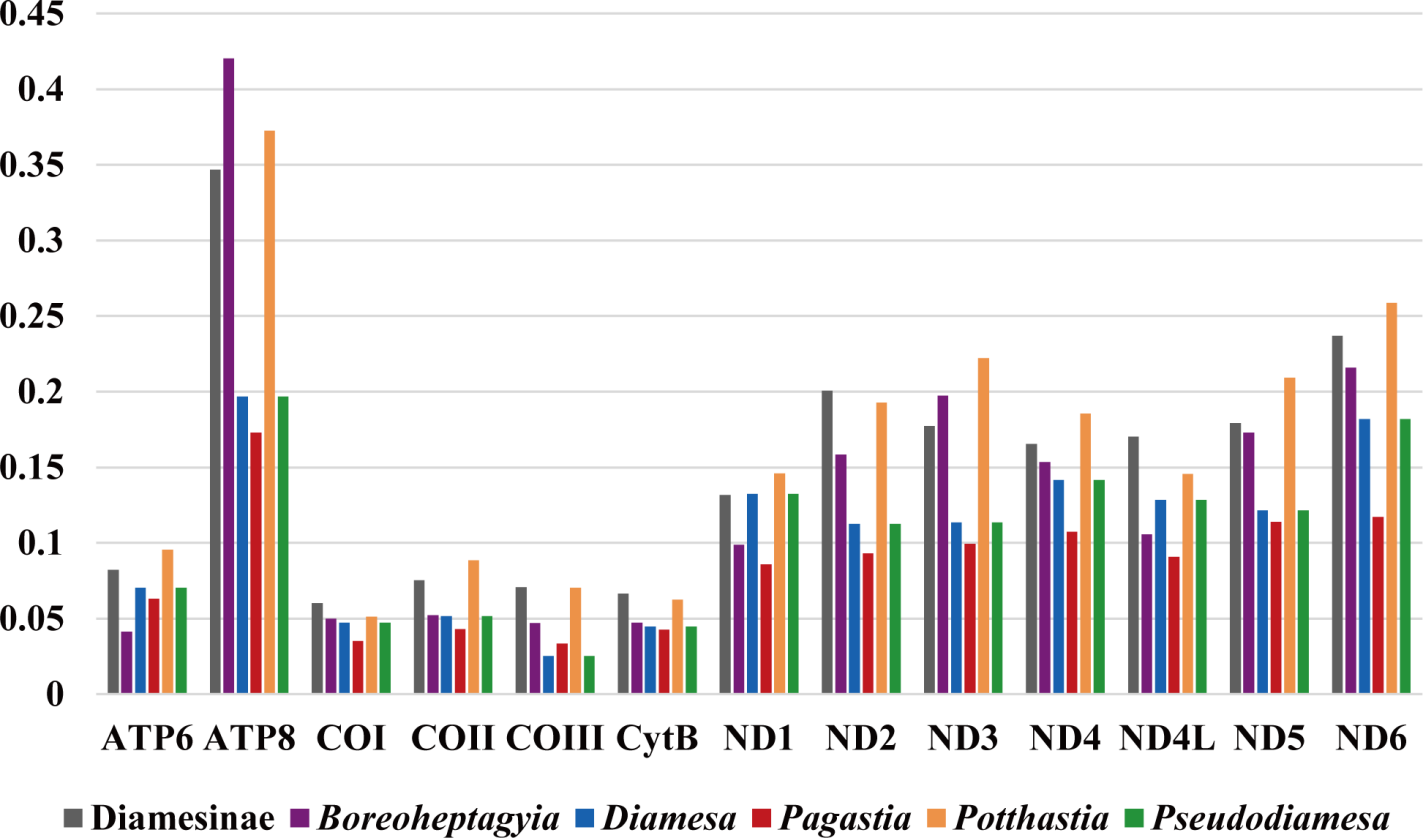
Evolution rate of each PCG of Diamesinae species. The *X*‐axis shows the names of PCGs and the *Y*‐axis shows the Ka/Ks value

For the entire Diamesinae, the ratio of Ka/Ks (ω) of all the 13 PCGs is less than 0.35, and the ATP8 exhibits the largest Ka/Ks value while the COI has the lowest Ka/Ks value (Figure 4, Table S3). To better understand the evolutionary pattern and the role of selection in Diamesinae species, the values of Ka/Ks were also calculated at congeneric level. The Ka/Ks value was quite heterogeneous at congeneric level. For individual genes, ATP6 showed a lower Ka/Ks value in *Boreoheptagyia*, ND1 and ND4L showed a lower Ka/Ks value in *Boreoheptagyia* and in *Pagastia*, and the remaining ten showed a lower Ka/Ks value in *Diamesa* and in *Pagastia* (Figure 4, Table S3). We also provided the Ka/Ks values of mitochondrial PCGs of Orthocladiinae and *Stenochironomu*s that we previous reported in Table S3, which are higher than that in Diamesinae.

Each mitogenome of Diamesinae contains 22 typical tRNA genes, with A+T content ranging from 74.0% to 77%. The nucleotide skew of tRNA genes among Diamesinae is consistent, exhibiting positive AT‐skew and negative GC‐skew (Figure 2, Figure S2). Both 12S and 16S rRNA genes transcribe from the minority strand (N‐strand). Among the mitogenomes of Diamesinae, the length of 12S rRNA gene varies from 794 to 815 bp, and the length of 16S rRNA gene varies from 1345 to 1374 bp (Table S2). The A+T content of 12S and 16S rRNA genes ranges from 76% to 78.6% and 80% to 82.8%, respectively. Both 12S and 16S rRNA genes exhibit positive GC‐skew in the mitogenomes of Diamesinae (Figure 2, Figure S2). A total of 21 mitogenomes in the present study have the complete control region, varying from 907 to 1309 bp (Table S2). The A+T content of the control region among the mitogenomes of Diamesinae ranges from 87.7% to 93.9% (Figure 2), extremely higher than the whole mitogenomes.

### 3.2 Phylogenetic relationships

**FIGURE 5:**
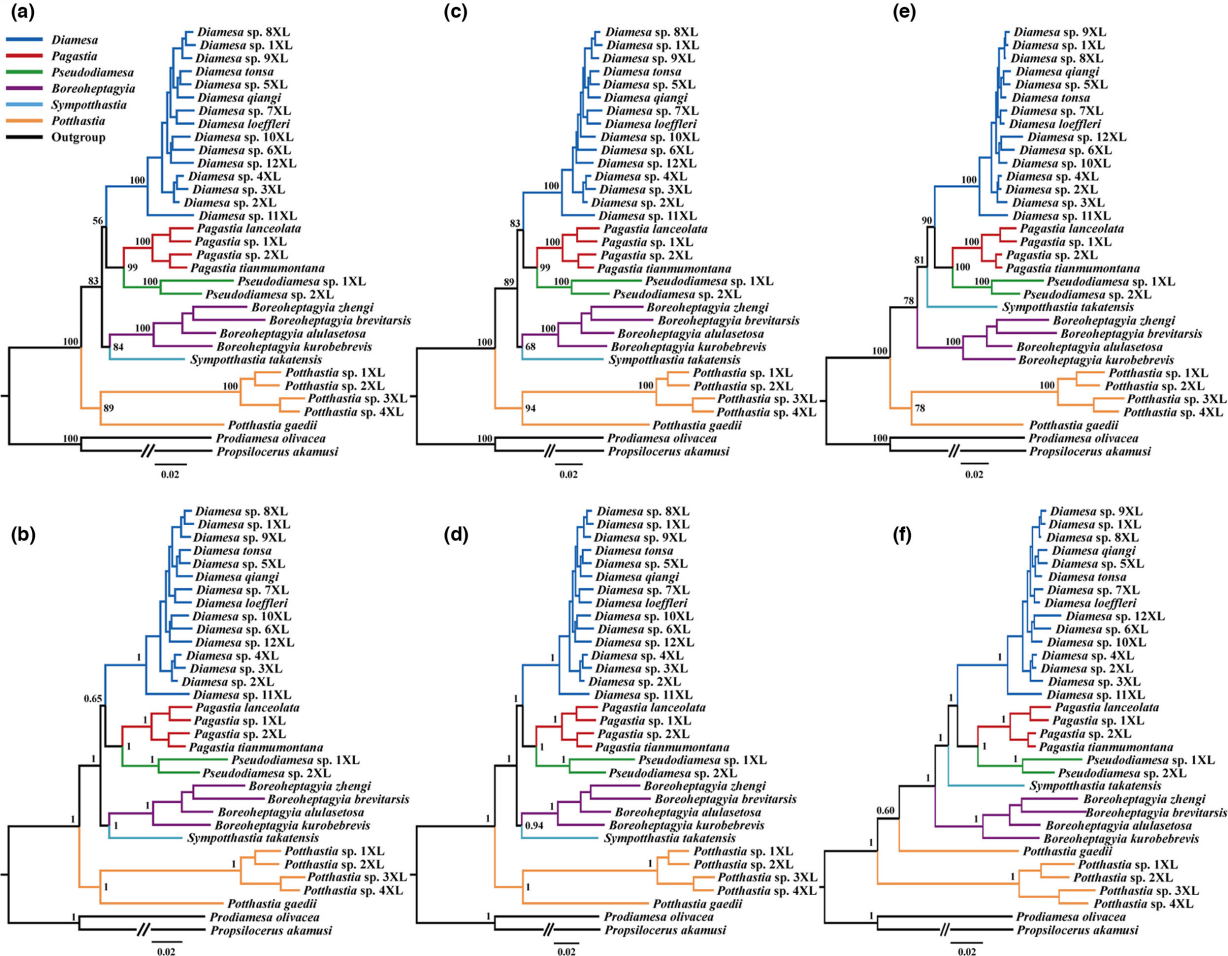
Phylogenetic relationships of Diamesinae inferred from mitogenomes. (a) ML tree obtained based on PCG12; (b) BI tree obtained based on PCG12; (c) ML tree obtained based on PCG12R; (d) BI tree obtained based on PCG12R; (e) ML tree obtained based on AA; (f) BI tree obtained based on AA. Numbers at the nodes are ML bootstrap values (a, c, e) and BI posterior probabilities (b, d, f)

Generally, six phylogenetic trees constructed by BI and ML analyses are similar in topology, only with the position of *Sympotthastia* was unstable (Figure 5). The monophyly of the Diamesinae is fully supported across all analyses using different datasets (Figure 5). Within the Diamesinae, two genera‐level topologies were inferred from three datasets: (i) (*Potthastia* + ((*Boreoheptagyia* +Sympotthastia) + (*Diamesa* + (*Pagastia* +Pseudodiamesa)))) was inferred from the PCG12 and PCG12R datasets; (ii) (*Potthastia* + (*Boreoheptagyia* + (*Sympotthastia* + (*Diamesa* + (*Pagastia* +Pseudodiamesa))))) was inferred from the AA dataset. The topology inferred from the AA dataset had the strongest nodal support. Based on three different datasets, Boreheptagyiini was deeply nested within Diamesini. The monophyly of *Boreoheptagyia*, *Diamesa*, *Pagastia*, *Potthastia*, and *Pseudodiamesa* was well supported by mitogenomes.

## 4 DISCUSSION

### 4.1 Mitogenome features

A total of 31 mitogenomes of Diamesinae are included in this study, of which 10 mitogenomes have incomplete control region by the highly gene duplication (Cameron, 2014; Zhang & Hewitt, 1997). The lengths of 21 complete mitogenomes of Diamesinae range from 15,913 bp to 16,411 bp due to the variation of the control region. The gene number and arrangement of these mitogenomes are conserved, and all genes arranged in the same order as the ancestral insect mitogenome (Clary & Wolstenholme, 1985). The nucleotide composition of the mitogenomes of Diamesinae is biased toward A+T, which is consistent with other published chironomid species (Beckenbach, 2012; Deviatiiarov et al., 2017; Zheng et al., 2021). The mitogenomes of Diamesinae exhibit positive or negative AT‐skew and negative GC‐skew, the nucleotide bias of these mitogenomes may be related to the asymmetric mutation processes during replication (Hassanin et al., 2005). Most PCGs of mitogenomes of Diamesinae terminated with complete termination codons, except ND4 and ND5 in a few mitogenomes, terminated with a single T that may be completed by post‐transcriptional polyadenylation (Ojala et al., 1981). The ratio of Ka/Ks (ω) is used to assess the evolutionary rate of PCGs in mitogenome (Cheng et al., 2018; Li et al., 2020). The lengths of rRNA genes are inconsistent among Diamesinae species, indicating a relatively high level of variation in these genes. The A+T content of the control region is significantly higher than the whole mitogenome and other regions in mitogenome in Diamesinae, indicating a strong A+T bias in this region.

### 4.2 Evolutionary rate

We compared the Ka/Ks value between Diamesinae and other subfamilies of Chironomidae. Previous studies reported the Ka/Ks values of mitochondrial PCGs of Orthocladiinae and *Stenochironomus* (Lin et al., 2022; Zheng et al., 2022), and the Ka/Ks values of each PCG in these chironomids are higher than that in Diamesinae (Table S3), indicating that the mitochondrial genomes of Diamesinae are under stronger purifying selection than other nonbiting midge species (Hurst, 2002). Mitochondrial genome played a central role in animal energy production, and stronger purification selection could enhance their conserved role in energy production (Hassanin et al., 2009; Yuan et al., 2020). The existence of stronger purifying selection in Diamesinae species may exhibit signs of adaptation to life at cold living conditions (high latitude and high altitude) (Makarchenko et al., 2017). Severe habitats generally accumulate more deleterious mutations, and the stronger purifying selection of mitochondrial PCGs in Diamesinae species may help against these deleterious mutations (Sarkar et al., 2020; Wang et al., 2019). In addition, Diamesinae species lives in the cold environment (Lencioni & Rossaro, 2005; Montagna et al., 2016; Sun et al., 2019) and have a small range of activities, which could lead to a lower metabolic rate. Given the correlation between purification selection and metabolic rate has been reported in several species (Chong & Mueller, 2013; Shen et al., 2009; Wang et al., 2019), we hypothesized that the stronger purifying selection in Diamesinae species may also be associated with metabolic requirement.

The evolutionary rate analyses of Diamesinae also provided new insights for the study of species delimitation. The evolutionary rate of COI was generally considered to be consistent with the evolutionary rate of the species itself, so it has been widely used in species delimitation and phylogenetic studies (Havill et al., 2021; Jones et al., 2021). However, for species with lower evolution rate of mitochondrial PCGs, COI barcodes sometimes failed to accurately define the species boundary of *Diamesa* (Montagna et al., 2016) (E. Stur, pers. comm.). The mitochondrial genome or individual genes with higher evolution rate may be better choices for species delimitation.

### 4.3 Phylogenetic analyses

Previous study has revealed that mitogenomes have poor phylogenetic signals at the subfamily level of Chironomidae (Zheng et al., 2021). However, our study reveals that the mitogenomes of Diamesinae are practically useful for phylogenetic inference. In our study, we applied a variety of strategies to explore the phylogenetic relationships of six genera of the Diamesinae using mitogenomic data, and confirmed the monophyly of Diamesinae. According to traditional morphological systematics, Boreheptagyiini could be separated from other tribes of Diamesinae by having distinct pubescence, low antennal ratio, and usual dorsocentral and prealar setae in adults (Brundin, 1966; Sæther, 1977; Serra‐Tosio, 1973). According the morphological phylogeny, tribe Boreheptagyiini is sister to Heptagyini + Lobodiamesini, and Diamesini is sister to Protanypini. However, in the dated molecular phylogeny of the Chironomidae (Cranston et al., 2012), Boreheptagyiini was not sampled, and only one Diamesa species was selected. Our result gives a new insight for the systematic status of Boreheptagyiini. Serra‐Tosio (1973) presented a simplistic analysis of the tribe Diamesini, indicating that the clade *Pagastia* + Pseudodiamesinae is sister to the clade (*Sympatthastia* + Potthastia) + (*Pseudokiefferiella* + (*Parapotthastia* + (*Onychodiamesa* + (*Diamesa* +Lappodiamesa)))). Willassen (2011) presented an unpublished study based on two mitochondrial genetic markers (COX2 and 16S) of all Diamesini genera except *Arctodiamesa* and *Sympotthastia* in the 18th International Symposium on Chironomidae, and found that the tribe Diamesini is not monophyletic unless *Potthastia* is removed, and *Boreoheptagyia* (and *Sasayusurika*) are sister to the remaining Diamesini. In our study, *Potthastia* is placed as the oldest of all Diamesinae genera studied here. Our result corresponds very well by Willassen (2011), supporting that *Potthastia* is not a Diamesini which is contradictory with traditional morphology‐based systematics. Moreover, the phylogenetic position of *Sympotthastia* remain unstable based on mitogenomic phylogeny. In general, missing taxa and lacking of informative genetic characters can give a wrong picture of phylogeny estimation (Xi et al., 2016). Therefore, to finally explore the evolutionary history of Diamesinae, a complete resolution will require a comprehensive taxa sampling with the most informative mitochondrial and nuclear markers.

## 5 CONCLUSION

In this study, we sequenced 30 mitogenomes representing 30 species of six genera of Boreheptagyiini and Diamesini by whole genome sequencing technologies, and did the first comparative analysis of mitogenome base composition and evolutionary history in Diamesinae. This study showed that mitogenomes of Diamesinae were conserved in structure, gene order, and nucleotide composition. All protein‐coding genes in Diamesinae were under stronger purifying selection than those of other nonbiting midge species, which may exhibit signs of adaptation to life at cold living conditions. Mitogenomes could provide new insight into evolutionary history of Diamesinae based on the dated molecular phylogeny.

## AUTHOR CONTRIBUTIONS

**Xiao‐Long Lin:** Data curation (lead); Investigation (equal); Methodology (equal); Software (equal); Writing – original draft (lead). **Zheng Liu:** Funding acquisition (lead); Writing – review & editing (equal). **Li‐Ping Yan:** Investigation (equal); Methodology (equal); Writing – review & editing (equal). **Xin Duan:** Formal analysis (equal); Software (equal). **Wen‐Jun Bu:** Supervision (equal); Validation (equal). **Xin‐Hua Wang:** Investigation (equal); Supervision (equal); Validation (equal). **Chen‐Guang Zheng:** Formal analysis (equal); Investigation (equal); Writing – original draft (equal); Writing – review & editing (equal).

## CONFLICT OF INTEREST

The authors declare that they have no conflicts of interest.

## Supporting information

Supplementary Material

## Data Availability

The new mitogenomes of *Boreoheptagyia alulasetosa*, *Boreoheptagyia brevitarsis*, *Boreoheptagyia kurobebrevis*, *Boreoheptagyia zhengi*, *Diamesa loeffleri*, *Diamesa qiangi*, *Diamesa* sp. 1XL, *Diamesa* sp. 2 XL, *Diamesa* sp. 3XL, *Diamesa* sp. 4XL, *Diamesa* sp. 5XL, *Diamesa* sp. 6XL, *Diamesa* sp. 7XL, *Diamesa* sp. 8XL, *Diamesa* sp. 9XL, *Diamesa* sp. 10XL, *Diamesa* sp. 11XL, *Diamesa* sp. 12XL, *Diamesa tonsa*, *Pagastia lanceolata*, *Pagastia* sp. 1XL, *Pagastia* sp. 2XL, *Pagastia tianmumontana*, *Potthastia gaedii*, *Potthastia* sp. 1XL, *Potthastia* sp. 2XL, *Potthastia* sp. 4XL, *Pseudodiamesa* sp. 1XL, *Pseudodiamesa* sp. 2XL, and *Sympotthastia takatensis* are deposited in GenBank of NCBI under accession numbers MZ043574, MZ043575, MZ043576, OM302508, MZ127838, MZ127839, MZ048035, MZ048036, MZ048037, MZ048038, MZ231027, MZ158293, MZ158294, MZ231028, MZ231029, MZ043577, MZ043578, MZ158295, MZ158292, OM302510, OM302507, OM302505, MZ231025, OM302504, OM302509, MZ064641, OM302503, MZ064643, OM302506, and MZ231026, respectively.

## References

[ece38957-bib-0001] Altschul, S. F. , Gish, W. , Miller, W. , Myers, E. W. , & Lipman, D. J. (1990). Basic local alignment search tool. Journal of Molecular Biology, 215(3), 403–410. 10.1016/S0022-2836(05)80360-2 2231712

[ece38957-bib-0002] Ashe, P. , & O'Connor, J. P. (2009). A world catalogue of Chironomidae (Diptera), Part 1: Buchonomyiinae, Chilenomyiinae, Podonominae, Aphroteniinae, Tanypodinae, Usambaromyiinae, Diamesinae, Prodiamesinae and Telmatogetoninae (445 pp). Irish Biogeographical Society.

[ece38957-bib-0003] Beckenbach, A. T. (2012). Mitochondrial genome sequences of Nematocera (lower Diptera): evidence of rearrangement following a complete genome duplication in a winter crane fly. Genome Biology and Evolution, 4(2), 89–101. 10.1093/gbe/evr131 22155689 PMC3269971

[ece38957-bib-0004] Bolger, A. M. , Lohse, M. , & Usadel, B. (2014). Trimmomatic: A flexible trimmer for Illumina sequence data. Bioinformatics, 30, 2114–2120. 10.1093/bioinformatics/btu170 24695404 PMC4103590

[ece38957-bib-0005] Boore, J. L. (1999). Animal mitochondrial genomes. Nucleic Acids Research, 27(8), 1767–1780. 10.1093/nar/27.8.1767 10101183 PMC148383

[ece38957-bib-0006] Brown, W. M. , George, M. , & Wilson, A. C. (1979). Rapid evolution of animal mitochondrial DNA. Proceedings of the National Academy of Sciences of the United States of America, 76(4), 1967–1971. 10.1073/pnas.76.4.1967 109836 PMC383514

[ece38957-bib-0007] Brundin, L. (1966). Transantarctic relationships and their significance, as evidenced by chironomid midges with a monograph of the subfamilies Podonominae and Aphroteniinae and the austral Heptagynae. Kongliga Svenska Vetenskaps Academiens nya Handlingar, 11, 1–472.

[ece38957-bib-0008] Cameron, S. L. (2014). Insect mitochondrial genomics: implications for evolution and phylogeny. Annual Review of Entomology, 59, 95–117. 10.1146/annurev-ento-011613-162007 24160435

[ece38957-bib-0009] Chen, J.‐Y. , Chang, Y.‐W. , Zheng, S.‐Z. , Lu, M.‐X. , & Du, Y.‐Z. (2018). Comparative analysis of the *Liriomyza chinensis* mitochondrial genome with other Agromyzids reveals conserved genome features. Scientific Reports, 8(1), 1–10. 10.1038/s41598-018-27213-7 29892001 PMC5995824

[ece38957-bib-0010] Cheng, Y. C. , Chen, M. Y. , Wang, J. F. , Liang, A. P. , & Lin, C. P. (2018). Some mitochondrial genes perform better for damselfly phylogenetics: Species‐and population‐level analyses of four complete mitogenomes of *Euphaea* sibling species. Systematic Entomology, 43(4), 702–715. 10.1111/syen.12299

[ece38957-bib-0011] Chong, R. A. , & Mueller, R. L. (2013). Low metabolic rates in salamanders are correlated with weak selective constraints on mitochondrial genes. Evolution, 67(3), 894–899. 10.1111/j.1558-5646.2012.01830.x 23461338

[ece38957-bib-0012] Clary, D. O. , & Wolstenholme, D. R. (1985). The mitochondrial DNA molecule of *Drosophila yakuba*: Nucleotide sequence, gene organization, and genetic code. Journal of Molecular Evolution, 22(3), 252–271. 10.1007/BF02099755 3001325

[ece38957-bib-0013] Condamine, F. L. , Nabholz, B. , Clamens, A.‐L. , Dupuis, J. R. , & Sperling, F. A. (2018). Mitochondrial phylogenomics, the origin of swallowtail butterflies, and the impact of the number of clocks in Bayesian molecular dating. Systematic Entomology, 43(3), 460–480. 10.1111/syen.12284

[ece38957-bib-0014] Crampton‐Platt, A. , Timmermans, M. J. , Gimmel, M. L. , Kutty, S. N. , Cockerill, T. D. , Vun Khen, C. , & Vogler, A. P. (2015). Soup to tree: The phylogeny of beetles inferred by mitochondrial metagenomics of a Bornean rainforest sample. Molecular Biology and Evolution, 32(9), 2302–2316. 10.1093/molbev/msv111 25957318 PMC4540967

[ece38957-bib-0015] Cranston, P. S. , Hardy, N. B. , & Morse, G. E. (2012). A dated molecular phylogeny for the Chironomidae (Diptera). Systematic Entomology, 37(1), 172–188. 10.1111/j.1365-3113.2011.00603.x

[ece38957-bib-0016] Cranston, P. S. , & Krosch, M. N. (2015). DNA sequences and austral taxa indicate generic synonymy of *Paratrichocladius* Santos‐Abreu with *Cricotopus* Wulp (Diptera: Chironomidae). Systematic Entomology, 40(4), 719–732. 10.1111/syen.12130

[ece38957-bib-0017] Curole, J. P. , & Kocher, T. D. (1999). Mitogenomics: Digging deeper with complete mitochondrial genomes. Trends in Ecology & Evolution, 14(10), 394–398. 10.1016/S0169-5347(99)01660-2 10481201

[ece38957-bib-0018] de Oliveira Aragão, A. , Neto, J. P. N. , Cruz, A. C. R. , Casseb, S. M. M. , Cardoso, J. F. , da Silva, S. P. , & Ishikawa, E. A. Y. (2019). Description and phylogeny of the mitochondrial genome of *Sabethes chloropterus*, *Sabethes glaucodaemon* and *Sabethes belisarioi* (Diptera: Culicidae). Genomics, 111(4), 607–611. 10.1016/j.ygeno.2018.03.016 29581026

[ece38957-bib-0019] Deviatiiarov, R. , Kikawada, T. , & Gusev, O. (2017). The complete mitochondrial genome of an anhydrobiotic midge *Polypedilum vanderplanki* (Chironomidae, Diptera). Mitochondrial DNA Part A, 28(2), 218–220. 10.3109/19401736.2015.1115849 26711887

[ece38957-bib-0020] Ekrem, T. (2003). Towards a phylogeny of *Tanytarsus* van der Wulp (Diptera: Chironomidae). Is morphology alone sufficient to reconstruct the genealogical relationship? Insect Systematics & Evolution, 34(2), 199–219.

[ece38957-bib-0021] Fang, X. , Li, X. , Lu, T. , Fu, J. , Shen, M. , Xiao, Y. , & Fu, Y. (2022). Complete mitochondrial genome of *Limnophyes minimus* (Diptera: Chironomidae). Mitochondrial DNA Part B, 7(1), 280–282. 10.1080/23802359.2022.2029604 35097216 PMC8797723

[ece38957-bib-0022] Grant, J. R. , & Stothard, P. (2008). The CGView Server: A comparative genomics tool for circular genomes. Nucleic Acids Research, 36(Suppl 2), 181–184. 10.1093/nar/gkn179 PMC2447734 18411202

[ece38957-bib-0023] Hassanin, A. , Leger, N. , & Deutsch, J. (2005). Evidence for multiple reversals of asymmetric mutational constraints during the evolution of the mitochondrial genome of Metazoa, and consequences for phylogenetic inferences. Systematic Biology, 54(2), 277–298. 10.1080/10635150590947843 16021696

[ece38957-bib-0024] Hassanin, A. , Ropiquet, A. , Couloux, A. , & Cruaud, C. (2009). Evolution of the mitochondrial genome in mammals living at high altitude: New insights from a study of the tribe Caprini (Bovidae, Antilopinae). Journal of Molecular Evolution, 68(4), 293–310. 10.1007/s00239-009-9208-7 19294454

[ece38957-bib-0025] Havill, N. P. , Griffin, B. P. , Andersen, J. C. , Foottit, R. G. , Justesen, M. J. , Caccone, A. , D'Amico, V. , & Elkinton, J. S. (2021). Species delimitation and invasion history of the balsam woolly adelgid, *Adelges* (*Dreyfusia*) *piceae* (Hemiptera: Aphidoidea: Adelgidae), species complex. Systematic Entomology, 46(1), 186–204. 10.1111/syen.12456

[ece38957-bib-0026] Hurst, L. D. (2002). The Ka/Ks ratio: Diagnosing the form of sequence evolution. Trends in Genetics, 18(9), 486. 10.1016/S0168-9525(02)02722-1 12175810

[ece38957-bib-0027] Jacobsen, M. W. , Hansen, M. M. , Orlando, L. , Bekkevold, D. , Bernatchez, L. , Willerslev, E. , & Gilbert, M. T. P. (2012). Mitogenome sequencing reveals shallow evolutionary histories and recent divergence time between morphologically and ecologically distinct European whitefish (*Coregonus* spp.). Molecular Ecology, 21(11), 2727–2742. 10.1111/j.1365-294X.2012.05561.x 22509795

[ece38957-bib-0028] Jiang, Y.‐W. , Zhao, Y.‐M. , & Lin, X.‐L. (2022). First report of the complete mitogenome of *Tanypus punctipennis* Meigen, 1818 (Diptera, Chironomidae) from Hebei Province, China. Mitochondrial DNA Part B, 7(1), 215–216. 10.1080/23802359.2021.2022544 35071760 PMC8774138

[ece38957-bib-0029] Jones, L. , Twyford, A. D. , Ford, C. R. , Rich, T. C. G. , Davies, H. , Forrest, L. L. , Hart, M. L. , McHaffie, H. , Brown, M. R. , Hollingsworth, P. M. , & Vere, N. (2021). Barcode UK: A complete DNA barcoding resource for the flowering plants and conifers of the United Kingdom. Molecular Ecology Resources, 21(6), 2050–2062. 10.1111/1755-0998.13388 33749162

[ece38957-bib-0030] Kang, Z. , Li, X. , & Yang, D. (2016). The complete mitochondrial genome of *Dixella* sp. (Diptera: Nematocera, Dixidae). Mitochondrial DNA Part A, 27(2), 1528–1529. 10.3109/19401736.2014.953123 25187169

[ece38957-bib-0031] Katoh, K. , & Standley, D. M. (2013). MAFFT multiple sequence alignment software version 7: Improvements in performance and usability. Molecular Biology and Evolution, 30(4), 772–780. 10.1093/molbev/mst010 23329690 PMC3603318

[ece38957-bib-0032] Kearse, M. , Moir, R. , Wilson, A. , Stones‐Havas, S. , Cheung, M. , Sturrock, S. , Buxton, S. , Cooper, A. , Markowitz, S. , Duran, C. , Thierer, T. , Ashton, B. , Meintjes, P. , & Drummond, A. (2012). Geneious Basic: An integrated and extendable desktop software platform for the organization and analysis of sequence data. Bioinformatics, 28(12), 1647–1649. 10.1093/bioinformatics/bts199 22543367 PMC3371832

[ece38957-bib-0033] Kelley, J. L. , Peyton, J. T. , Fiston‐Lavier, A.‐S. , Teets, N. M. , Yee, M.‐C. , Johnston, J. S. , Bustamante, C. D. , Lee, R. E. , & Denlinger, D. L. (2014). Compact genome of the Antarctic midge is likely an adaptation to an extreme environment. Nature Communications, 5, 4611. 10.1038/ncomms5611 PMC4164542 25118180

[ece38957-bib-0034] Kim, S. , Kim, H. , & Shin, S. C. (2016). Complete mitochondrial genome of the Antarctic midge *Parochlus steinenii* (Diptera: Chironomidae). Mitochondrial DNA Part A, 27(5), 3475–3476. 10.3109/19401736.2015.1066355 26642812

[ece38957-bib-0035] Kong, F.‐Q. , Zhao, Y.‐C. , Chen, J.‐L. , & Lin, X.‐L. (2021). First report of the complete mitogenome of *Microchironomus tabarui* Sasa, 1987 (Diptera, Chironomidae) from Hebei Province, China. Mitochondrial DNA Part B, 6(10), 2845–2846. 10.1080/23802359.2021.1970638 34514149 PMC8425763

[ece38957-bib-0036] Krosch, M. , & Cranston, P. S. (2013). Not drowning, (hand) waving? Molecular phylogenetics, biogeography and evolutionary tempo of the ‘Gondwanan’ midge Stictocladius Edwards (Diptera: Chironomidae). Molecular Phylogenetics and Evolution, 68(3), 595–603. 10.1016/j.ympev.2013.04.006 23608128

[ece38957-bib-0037] Kumar, S. , Stecher, G. , Li, M. , Knyaz, C. , & Tamura, K. (2018). MEGA X: Molecular evolutionary genetics analysis across computing platforms. Molecular Biology and Evolution, 35(6), 1547–1549. 10.1093/molbev/msy096 29722887 PMC5967553

[ece38957-bib-0038] Lanfear, R. , Frandsen, P. B. , Wright, A. M. , Senfeld, T. , & Calcott, B. (2017). PartitionFinder 2: New methods for selecting partitioned models of evolution for molecular and morphological phylogenetic analyses. Molecular Biology and Evolution, 34(3), 772–773. 10.1093/molbev/msw260 28013191

[ece38957-bib-0039] Lei, T. , Song, C. , Zhu, X.‐D. , Xu, B.‐Y. , & Qi, X. (2021). The complete mitochondrial genome of a non‐biting midge *Polypedilum unifascium* (Tokunaga, 1938) (Diptera: Chironomidae). Mitochondrial DNA Part B, 6(8), 2212–2213. 10.1080/23802359.2021.1945977 34263051 PMC8259822

[ece38957-bib-0040] Lencioni, V. , Rodriguez‐Prieto, A. , & Allegrucci, G. (2021). Congruence between molecular and morphological systematics of Alpine non‐biting midges (Chironomidae, Diamesinae). Zoologica Scripta, 50(4), 455–472. 10.1111/zsc.12480

[ece38957-bib-0041] Lencioni, V. , & Rossaro, B. (2005). Microdistribution of chironomids (Diptera: Chironomidae) in Alpine streams: an autoecological perspective. Hydrobiologia, 533(1), 61–76. 10.1007/s10750-004-2393-x

[ece38957-bib-0042] Li, X.‐Y. , Yan, L.‐P. , Pape, T. , Gao, Y.‐Y. , & Zhang, D. (2020). Evolutionary insights into bot flies (Insecta: Diptera: Oestridae) from comparative analysis of the mitochondrial genomes. International Journal of Biological Macromolecules, 149, 371–380. 10.1016/j.ijbiomac.2020.01.249 31991213

[ece38957-bib-0043] Lin, X.‐L. , Chang, T. , Yan, C.‐C. , Wang, B. , & Liu, W.‐B. (2021). Redescription of *Diamesa loeffleri* Reiss, 1968 (Diptera, Chironomidae) and new record from China. Annales Zoologici Fennici, 58(1–3), 109–113. 10.5735/086.058.0110

[ece38957-bib-0044] Lin, X.‐L. , Stur, E. , & Ekrem, T. (2018). Molecular phylogeny and temporal diversification of *Tanytarsus* van der Wulp (Diptera: Chironomidae) support generic synonymies, a new classification and centre of origin. Systematic Entomology, 43, 659–677. 10.1111/syen.12292

[ece38957-bib-0045] Lin, X.‐L. , Yu, H.‐J. , Wang, X.‐H. , Bu, W.‐J. , Wang, X.‐H. , Yan, C.‐C. , & Liu, W.‐B. (2021). New or little‐known *Boreoheptagyia* (Diptera: Chironomidae) in China inferred from morpholgy and DNA barcodes. ZooKeys, 1040, 187–200. 10.3897/zookeys.1040.66527 34135660 PMC8178290

[ece38957-bib-0046] Lin, X.‐L. , Zhao, Y.‐M. , Yan, L.‐P. , Liu, W.‐B. , Bu, W.‐J. , Wang, X.‐H. , & Zheng, C.‐G. (2022). Mitogenomes provide new insights into the evolutionary history of Prodiamesinae (Diptera: Chironomidae). Zoologica Scripta, 51(1), 119–132. 10.1111/zsc.12516

[ece38957-bib-0047] Makarchenko, E. A. , Endo, K. , Wu, J.‐Y. , & Wang, X.‐H. (2008). A review of *Boreoheptagyia* Brundin, 1966 (Chironomidae: Diamesinae) from East Asia and bordering territories, with the description of five new species. Zootaxa, 1817, 1–17. 10.11646/zootaxa.1817.1.1

[ece38957-bib-0048] Makarchenko, E. A. , Semenchenko, A. A. , & Palatov, D. M. (2017). Review of subfamily Diamesinae (Diptera, Chironomidae) from Tien Shan and Pamir mountains. Paper presented at the 20th International Symposium on Chironomidae. Abstract Book of the 20th International Symposium on Chironomidae MUSE—Museo delle Scienze, Trento, Italy, 2–8 July 2017.

[ece38957-bib-0049] Makarchenko, E. A. , Semenchenko, A. A. , & Palatov, D. M. (2021). New species and findings of *Pagastia* Oliver (Diptera: Chironomidae: Diamesinae) from Central Asia, with DNA barcoding of known species of the genus. Zootaxa, 4951(3), 559–570. 10.11646/zootaxa.4951.3.8 33903395

[ece38957-bib-0050] Makarchenko, E. A. , & Wang, X.‐H. (2017). *Pagastia tianmumontana* sp. n.‐a new species of chironomids (Diptera: Chironomidae: Diamesinae) from South China. Far Eastern Entomologist, 336, 13–15.

[ece38957-bib-0051] Miao, X. , Huang, J. , Menzel, F. , Wang, Q. , Wei, Q. , Lin, X.‐L. , & Wu, H. (2020). Five mitochondrial genomes of black fungus gnats (Sciaridae) and their phylogenetic implications. International Journal of Biological Macromolecules, 150, 200–205. 10.1016/j.ijbiomac.2020.01.271 32004603

[ece38957-bib-0052] Montagna, M. , Mereghetti, V. , Lencioni, V. , & Rossaro, B. (2016). Integrated taxonomy and DNA barcoding of alpine midges (Diptera: Chironomidae). PLoS One, 11(3), e0149673. 10.1371/journal.pone.0149673 26938660 PMC4777558

[ece38957-bib-0053] Moubayed‐Breil, J. , & Orsini, A. (2016). On the genus *Potthastia* Kieffer, 1922 from Corsica and continental France with description of three new species [Diptera, Chironomidae, Diamesinae]. Ephemera, 17, 1–36.

[ece38957-bib-0054] Nguyen, L.‐T. , Schmidt, H. A. , Von Haeseler, A. , & Minh, B. Q. (2015). IQ‐TREE: a fast and effective stochastic algorithm for estimating maximum‐likelihood phylogenies. Molecular Biology and Evolution, 32(1), 268–274. 10.1093/molbev/msu300 25371430 PMC4271533

[ece38957-bib-0055] Ojala, D. , Montoya, J. , & Attardi, G. (1981). tRNA punctuation model of RNA processing in human mitochondria. Nature, 290(5806), 470–474. 7219536 10.1038/290470a0

[ece38957-bib-0056] Oliver, D. R. (1983). The larvae of Diamesinae (Diptera: Chironomidae) of the Holarctic region– Keys and diagnoses. Entomologica Scandinavica, Supplement, 19, 115–138.

[ece38957-bib-0057] Oliver, D. (1989). The adult males of Diamesinae (Diptera. Chironomidae) of the Holarctic region – keys and diagnoses. Entomologica Scandinavica, Supplement, 34, 129–154.

[ece38957-bib-0058] Park, K. , Jo, H. , Choi, B. , & Kwak, I.‐S. (2020). Complete mitochondrial genome of *Stictochironomus akizukii* (Tokunaga) (Chironomidae, Diptera) assembled from next‐generation sequencing data. Mitochondrial DNA Part B, 5(3), 2310–2311. 10.1080/23802359.2020.1750320 33457771 PMC7782153

[ece38957-bib-0059] Peng, Y. , Leung, H. C. , Yiu, S.‐M. , & Chin, F. Y. (2012). IDBA‐UD: A *de novo* assembler for single‐cell and metagenomic sequencing data with highly uneven depth. Bioinformatics, 28(11), 1420–1428. 10.1093/bioinformatics/bts174 22495754

[ece38957-bib-0060] Qi, X. , Lin, X.‐L. , Ekrem, T. , Beutel, R. G. , Song, C. , Orlov, I. , Chen, C.‐T. , & Wang, X.‐H. (2019). A new surface gliding species of Chironomidae: An independent invasion of marine environments and its evolutionary implications. Zoologica Scripta, 48(1), 81–92. 10.1111/zsc.12331

[ece38957-bib-0061] Ramakodi, M. P. , Singh, B. , Wells, J. D. , Guerrero, F. , & Ray, D. A. (2015). A 454 sequencing approach to dipteran mitochondrial genome research. Genomics, 105(1), 53–60. 10.1016/j.ygeno.2014.10.014 25451744

[ece38957-bib-0062] Rambaut, A. , Drummond, A. J. , Xie, D. , Baele, G. , & Suchard, M. A. (2018). Posterior summarization in Bayesian phylogenetics using Tracer 1.7. Systematic Biology, 67(5), 901–904. 10.1093/sysbio/syy032 29718447 PMC6101584

[ece38957-bib-0063] Reiss, F. (1968). Neue Chironomiden‐Arten (Diptera) aus Nepal. Khumbu Himal, 3, 55–73.

[ece38957-bib-0064] Ronquist, F. , Teslenko, M. , van der Mark, P. , Ayres, D. L. , Darling, A. , Höhna, S. , Larget, B. , Liu, L. , Suchard, M. A. , & Huelsenbeck, J. P. (2012). MrBayes 3.2: Efficient Bayesian phylogenetic inference and model choice across a large model space. Systematic Biology, 61(3), 539–542. 10.1093/sysbio/sys029 22357727 PMC3329765

[ece38957-bib-0065] Rozas, J. , Ferrer‐Mata, A. , Sánchez‐DelBarrio, J. C. , Guirao‐Rico, S. , Librado, P. , Ramos‐Onsins, S. E. , & Sánchez‐Gracia, A. (2017). DnaSP 6: DNA sequence polymorphism analysis of large data sets. Molecular Biology and Evolution, 34(12), 3299–3302. 10.1093/molbev/msx248 29029172

[ece38957-bib-0066] Sæther, O. A. (1969). Some Nearctic Podonominae, Diamesinae, and Orthocladiinae (Diptera: Chironomidae). Bulletin of the Fisheries Research Board of Canada, 170, 1–154.

[ece38957-bib-0067] Sæther, O. A. (1977). Female genitalia in Chironomidae and other Nematocera: Morphology, phylogenies, keys. Bulletin of the Fisheries Research Board of Canada, 197, 1–209.

[ece38957-bib-0068] Sæther, O. A. (2000). Phylogeny of the subfamilies of Chironomidae (Diptera). Systematic Entomology, 25(3), 393–403. 10.1046/j.1365-3113.2000.00111.x

[ece38957-bib-0069] Sarkar, I. , Dey, P. , Sharma, S. K. , Ray, S. D. , Kochiganti, V. H. S. , Singh, R. , Pramod, P. , & Singh, R. P. (2020). Turdoides affinis mitogenome reveals the translational efficiency and importance of NADH dehydrogenase complex‐I in the Leiothrichidae family. Scientific Reports, 10(1), 1–11. 10.1038/s41598-020-72674-4 33004841 PMC7530654

[ece38957-bib-0070] Serra‐Tosio, B. (1973). Ecologie et biogéographie des Diamesini d’Europe (Diptera, Chironomidae). Travaux du Laboratoire d’hydrobiologie et de pisciculture de l’université de Grenoble, 63, 5–175.

[ece38957-bib-0071] Shen, Y.‐Y. , Shi, P. , Sun, Y.‐B. , & Zhang, Y.‐P. (2009). Relaxation of selective constraints on avian mitochondrial DNA following the degeneration of flight ability. Genome Research, 19(10), 1760–1765. 10.1101/gr.093138.109 19617397 PMC2765268

[ece38957-bib-0072] Silva, F. L. , Ekrem, T. , & Fonseca‐Gessner, A. A. (2015). Out of South America: phylogeny of non‐biting midges in the genus *Labrundinia* suggests multiple dispersal events to Central and North America. Zoologica Scripta, 44(1), 59–71. 10.1111/Zsc.12089

[ece38957-bib-0073] Sun, B.‐J. , Lin, X.‐L. , Wang, X.‐H. , & Makarchenko, E. A. (2019). New or little‐known Diamesinae (Diptera: Chironomidae) from Oriental China. Zootaxa, 4571(4), 544–550. 10.11646/zootaxa.4571.4.6 31715794

[ece38957-bib-0074] Tang, L. , Yan, L. , Gao, Y. , & Zhang, D. (2019). First report of mitochondrial genome from the subfamily Bengaliinae (Diptera: Calliphoridae). Mitochondrial DNA Part B, 4(1), 1560–1561. 10.1080/23802359.2019.1601037

[ece38957-bib-0075] Tang, P. , Zhu, J.‐C. , Zheng, B.‐Y. , Wei, S.‐J. , Sharkey, M. , Chen, X.‐X. , & Vogler, A. P. (2019). Mitochondrial phylogenomics of the Hymenoptera. Molecular Phylogenetics and Evolution, 131, 8–18. 10.1016/j.ympev.2018.10.040 30399430

[ece38957-bib-0076] Vaidya, G. , Lohman, D. J. , & Meier, R. (2011). SequenceMatrix: concatenation software for the fast assembly of multi‐gene datasets with character set and codon information. Cladistics, 27(2), 171–180. 10.1111/j.1096-0031.2010.00329.x 34875773

[ece38957-bib-0077] Wang, Q. , Huang, J. , & Wu, H. (2021). Mitogenomes provide insights into the phylogeny of Mycetophilidae (Diptera: Sciaroidea). Gene, 783, 145564. 10.1016/j.gene.2021.145564 33711408

[ece38957-bib-0078] Wang, X. , Zhang, Y. , Zhang, H. , Qin, G. , & Lin, Q. (2019). Complete mitochondrial genomes of eight seahorses and pipefishes (Syngnathiformes: Syngnathidae): Insight into the adaptive radiation of syngnathid fishes. BMC Evolutionary Biology, 19(1), 1–11. 10.1186/s12862-019-1430-3 31185889 PMC6560779

[ece38957-bib-0079] Willassen, E. (2011). Phylogeny of Diamesinae inferred from mtDNA sequences. Paper presented at the 18th International Symposium on Chironomidae. Scientific Program and Abstracts (p. 50). https://www.ntnu.no/c/document_library/get_file?uuid=515831d0‐116b‐4dd6‐a5d7‐03de9f235816&groupId=10476

[ece38957-bib-0080] Wolstenholme, D. R. (1992). Animal mitochondrial DNA: Structure and evolution. International Review of Cytology, 141, 173–216. 10.1016/S0074-7696(08)62066-5 1452431

[ece38957-bib-0081] Xi, Z. X. , Liu, L. , & Davis, C. C. (2016). The impact of missing data on species tree estimation. Molecular Biology and Evolution, 33(3), 838–860. 10.1093/molbev/msv266 26589995

[ece38957-bib-0082] Xia, X. H. (2013). DAMBE5: A comprehensive software package for data analysis in molecular biology and evolution. Molecular Biology and Evolution, 30(7), 1720–1728. 10.1093/molbev/mst064 23564938 PMC3684854

[ece38957-bib-0083] Yan, L. , Pape, T. , Elgar, M. A. , Gao, Y. , & Zhang, D. (2019). Evolutionary history of stomach bot flies in the light of mitogenomics. Systematic Entomology, 44(4), 797–809. 10.1111/syen.12356

[ece38957-bib-0084] Yan, L. , Xu, W. , Zhang, D. , & Li, J. (2021). Comparative analysis of the mitochondrial genomes of flesh flies and their evolutionary implication. International Journal of Biological Macromolecules, 174, 385–391. 10.1016/j.ijbiomac.2021.01.188 33529628

[ece38957-bib-0085] Yuan, M.‐L. , Zhang, L.‐J. , Zhang, Q.‐L. , Zhang, L. I. , Li, M. , Wang, X.‐T. , Feng, R.‐Q. , & Tang, P.‐A. (2020). Mitogenome evolution in ladybirds: Potential association with dietary adaptation. Ecology and Evolution, 10(2), 1042–1053. 10.1002/ece3.5971 32015863 PMC6988538

[ece38957-bib-0086] Zhang, D.‐X. , & Hewitt, G. M. (1997). Insect mitochondrial control region: A review of its structure, evolution and usefulness in evolutionary studies. Biochemical Systematics and Ecology, 25(2), 99–120. 10.1016/S0305-1978(96)00042-7

[ece38957-bib-0087] Zhang, D. , Yan, L. , Zhang, M. , Chu, H. , Cao, J. , Li, K. , Hu, D. , & Pape, T. (2016). Phylogenetic inference of calyptrates, with the first mitogenomes for Gasterophilinae (Diptera: Oestridae) and Paramacronychiinae (Diptera: Sarcophagidae). International Journal of Biological Sciences, 12(5), 489–504. 10.7150/ijbs.12148 27019632 PMC4807417

[ece38957-bib-0088] Zhang, Q. , Xu, W. , Peng, K. , Zou, L. , Li, Y. , Chen, Y. , Cai, Y. , & Gong, Z. (2019). The complete mitochondrial genome of *Propsilocerus akamusi* (Diptera, Chironomidae). Mitochondrial DNA Part B, 4(2), 3983–3984. 10.1080/23802359.2019.1688703 33366281 PMC7707779

[ece38957-bib-0089] Zhang, X. , Kang, Z. , Ding, S. , Wang, Y. , Borkent, C. , Saigusa, T. , & Yang, D. (2019). Mitochondrial genomes provide insights into the phylogeny of Culicomorpha (Insecta: Diptera). International Journal of Molecular Sciences, 20(3), 747. 10.3390/ijms20030747 PMC6387087 30754618

[ece38957-bib-0090] Zhang, X. , Yang, D. , & Kang, Z. (2022). New data on the mitochondrial genome of Nematocera (lower Diptera): Features, structures and phylogenetic implications. Zoological Journal of the Linnean Society, zlac012. 10.1093/zoolinnean/zlac012

[ece38957-bib-0091] Zheng, C.‐G. , Liu, Z. , Zhao, Y.‐M. , Wang, Y. , Bu, W.‐J. , Wang, X.‐H. , & Lin, X.‐L. (2022). First report on mitochondrial gene rearrangement in non‐biting midges, revealing a Synapomorphy in *Stenochironomus* Kieffer (Diptera: Chironomidae). Insects, 13(2), 115. 10.3390/insects13020115 35206689 PMC8875173

[ece38957-bib-0092] Zheng, C. , Ye, Z. , Zhu, X. , Zhang, H. , Dong, X. , Chen, P. , & Bu, W. (2020). Integrative taxonomy uncovers hidden species diversity in the rheophilic genus *Potamometra* (Hemiptera: Gerridae). Zoologica Scripta, 49(2), 174–186. 10.1111/zsc.12401

[ece38957-bib-0093] Zheng, C.‐G. , Zhu, X.‐X. , Yan, L.‐P. , Yao, Y. , Bu, W.‐J. , Wang, X.‐H. , & Lin, X.‐L. (2021). First complete mitogenomes of Diamesinae, Orthocladiinae, Prodiamesinae, Tanypodinae (Diptera: Chironomidae) and their implication in phylogenetics. PeerJ, 9, e11294. 10.7717/peerj.11294 33996279 PMC8106913

