# Supplementary material for: Mitogenomes provide new insights of evolutionary history of Boreheptagyiini and Diamesini (Diptera: Chironomidae: Diamesinae)
Source: Ecol Evol. 2022 May 24;12(5):e8957. doi: 10.1002/ece3.8957 (PMC9130564; doi:10.1002/ece3.8957)
Supplement: Supplementary file 1 — Supplementary Material [file ECE3-12-e8957-s001.docx]

**Supporting information**

**Table S1.** The best model for each partition of the three datasets.

| Datasets | Partition names | Best model |
| --- | --- | --- |
| PCG12 | ATP6, CO1, CO2, CO3, CytB; | TIM+I+G |
|  | ATP6, ND1, ND2, ND3, ND4, ND4L, ND5, ND6; | GTR+I+G |
| PCG12R | ATP6, CO1, CO2, CO3, CytB; | TIM +I+G |
|  | ATP6, ND1, ND2, ND3, ND4, ND4L, ND5, ND6; | GTR+I+G |
|  | 12S, 16S; | TVM+I+G |
| AA | ATP6, CO1, CO2, CO3, CytB, ND4L; | MTART+I+G |
|  | ATP6, ND1, ND2, ND3, ND4, ND5, ND6; | MTART+I+G |

**Table S2.** Length of mitochondrial genes of Diamesinae species.

| Species | Gene length (bp) | | | |
| --- | --- | --- | --- | --- |
|  | Whole genome | Cotrol region | 12S rRNA | 16S rRNA |
| *Boreoheptagyia alulasetosa* | 16,411 | 1261 | 802 | 1359 |
| *Boreoheptagyia brevitarsis* | 16,086 | 1029 | 805 | 1368 |
| *Boreoheptagyia kurobebrevis* | 16,409 | 1309 | 803 | 1367 |
| *Boreoheptagyia zhengi* | 16,180 | 1068 | 801 | 1368 |
| *Diamesa loffleri* | 16,113 | 1046 | 805 | 1374 |
| *Diamesa qiangi* | 15,894 | 843 | 804 | 1371 |
| *Diamesa* sp. 10XL | 16,131 | 1069 | 803 | 1373 |
| *Diamesa* sp. 11XL | 16,195 | 1129 | 803 | 1373 |
| *Diamesa* sp. 1XL | 16,060 | 1019 | 803 | 1373 |
| *Diamesa* sp. 2 XL | 16,152 | 1100 | 803 | 1373 |
| *Diamesa* sp. 3XL | 16,183 | 1140 | 805 | 1373 |
| *Diamesa* sp. 4XL | 16,153 | 1112 | 803 | 1372 |
| *Diamesa* sp. 5XL | 16,012 | 998 | 803 | 1345 |
| *Diamesa* sp. 6XL | 16,186 | 1129 | 803 | 1373 |
| *Diamesa* sp. 7XL | 15,952 | 881 | 803 | 1373 |
| *Diamesa* sp. 12XL | 16,182 | 1111 | 803 | 1373 |
| *Diamesa* sp. 8XL | 15,923 | 884 | 803 | 1373 |
| *Diamesa* sp. 9XL | 15,725 | 673 | 803 | 1373 |
| *Diamesa tonsa* | 16,152 | 1109 | 807 | 1372 |
| *Pagastia lanceolata* | 16,134 | 1083 | 815 | 1366 |
| *Pagastia* sp. 1XL | 16,160 | 1059 | 808 | 1367 |
| *Pagastia* sp. 2XL | 16,165 | 1124 | 806 | 1365 |
| *Pagastia tianmumontana* | 16,143 | 1053 | 807 | 1365 |
| *Potthastia gaddei* | 15,285 | 275 | 794 | 1360 |
| *Potthastia* sp. 1XL | 15,991 | 943 | 799 | 1355 |
| *Potthastia* sp. 2XL | 16,016 | 993 | 797 | 1354 |
| *Potthastia* sp. 3XL | 15,913 | 993 | 797 | 1354 |
| *Potthastia* sp. 4XL | 15,946 | 955 | 794 | 1353 |
| *Pseudodiamesa* sp. 1XL | 16,012 | 907 | 804 | 1365 |
| *Pseudodiamesa* sp. 2XL | 16,033 | 961 | 809 | 1367 |
| *Sympotthastia takatensis* | 16,258 | 1174 | 807 | 1367 |

**Table S3.** Evolution rate of each PCG of Chironomidae species.

|  | Orthocladiinae | *Stenochironomus* | Diamesinae | *Boreoheptagyia* | *Diamesa* | *Pagastia* | *Potthastia* | *Pseudodiamesa* |
| --- | --- | --- | --- | --- | --- | --- | --- | --- |
| ATP6 | 0.181 | 0.267 | 0.082 | 0.041 | 0.070 | 0.063 | 0.095 | 0.070 |
| ATP8 | 0.552 | 0.466 | 0.347 | 0.420 | 0.197 | 0.173 | 0.372 | 0.197 |
| COI | 0.093 | 0.109 | 0.060 | 0.050 | 0.047 | 0.035 | 0.051 | 0.047 |
| COII | 0.100 | 0.210 | 0.075 | 0.052 | 0.052 | 0.043 | 0.089 | 0.052 |
| COIII | 0.115 | 0.179 | 0.071 | 0.047 | 0.025 | 0.033 | 0.070 | 0.025 |
| CytB | 0.120 | 0.210 | 0.066 | 0.047 | 0.045 | 0.043 | 0.062 | 0.045 |
| ND1 | 0.210 | 0.398 | 0.132 | 0.099 | 0.132 | 0.086 | 0.146 | 0.132 |
| ND2 | 0.264 | 0.430 | 0.201 | 0.158 | 0.113 | 0.093 | 0.193 | 0.113 |
| ND3 | 0.259 | 0.304 | 0.177 | 0.197 | 0.114 | 0.099 | 0.222 | 0.114 |
| ND4 | 0.278 | 0.414 | 0.165 | 0.154 | 0.142 | 0.107 | 0.185 | 0.142 |
| ND4L | 0.215 | 0.380 | 0.170 | 0.106 | 0.128 | 0.091 | 0.146 | 0.128 |
| ND5 | 0.290 | 0.400 | 0.179 | 0.173 | 0.122 | 0.114 | 0.209 | 0.122 |
| ND6 | 0.368 | 0.538 | 0.237 | 0.216 | 0.182 | 0.117 | 0.259 | 0.182 |


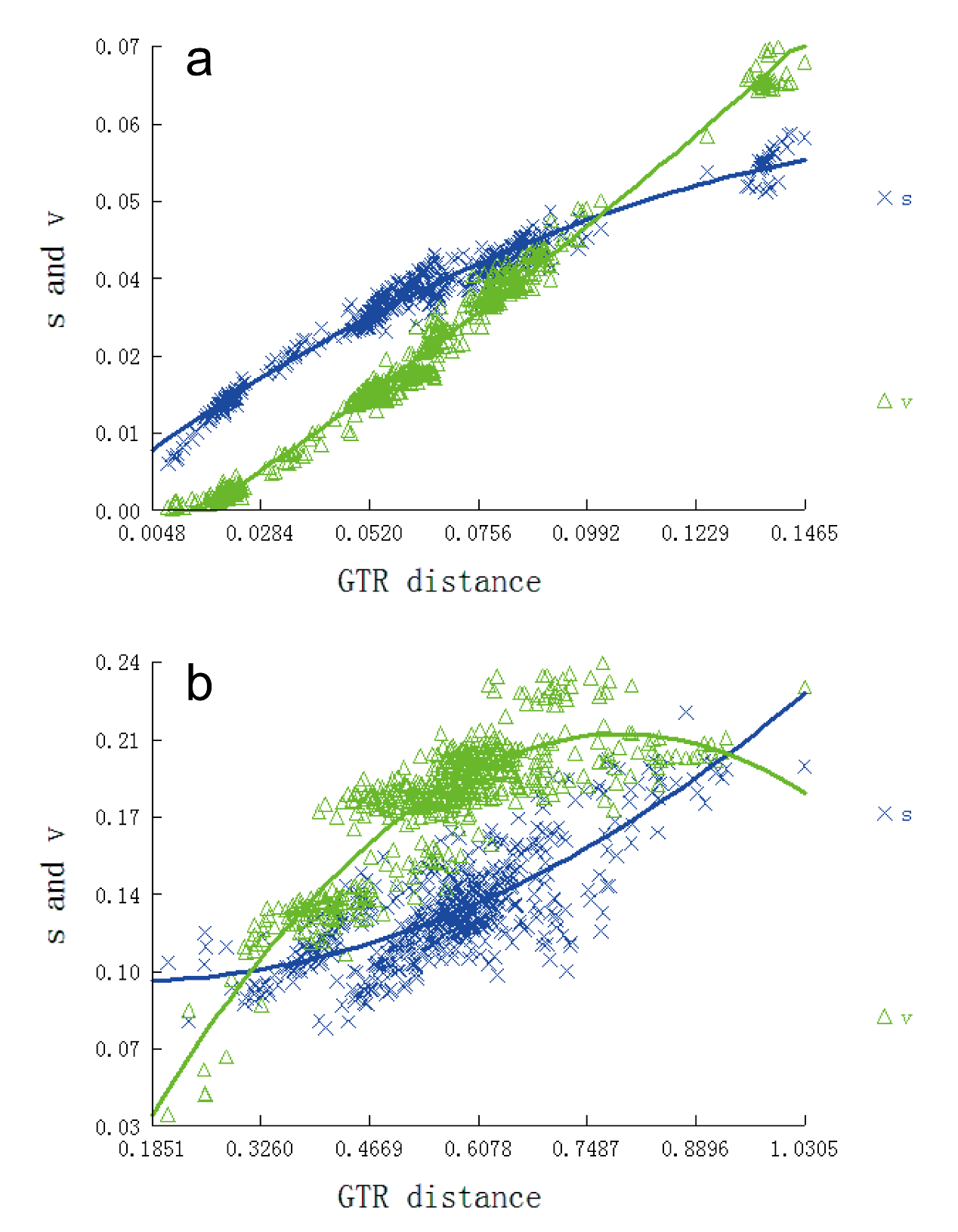


**Figure S1.** Nucleotide substitution saturation plots of PCGs. (a) the 1st and 2nd codon positions; (b) the 3rd codon position. Plots in blue and green indicate transition and transversion respectively.


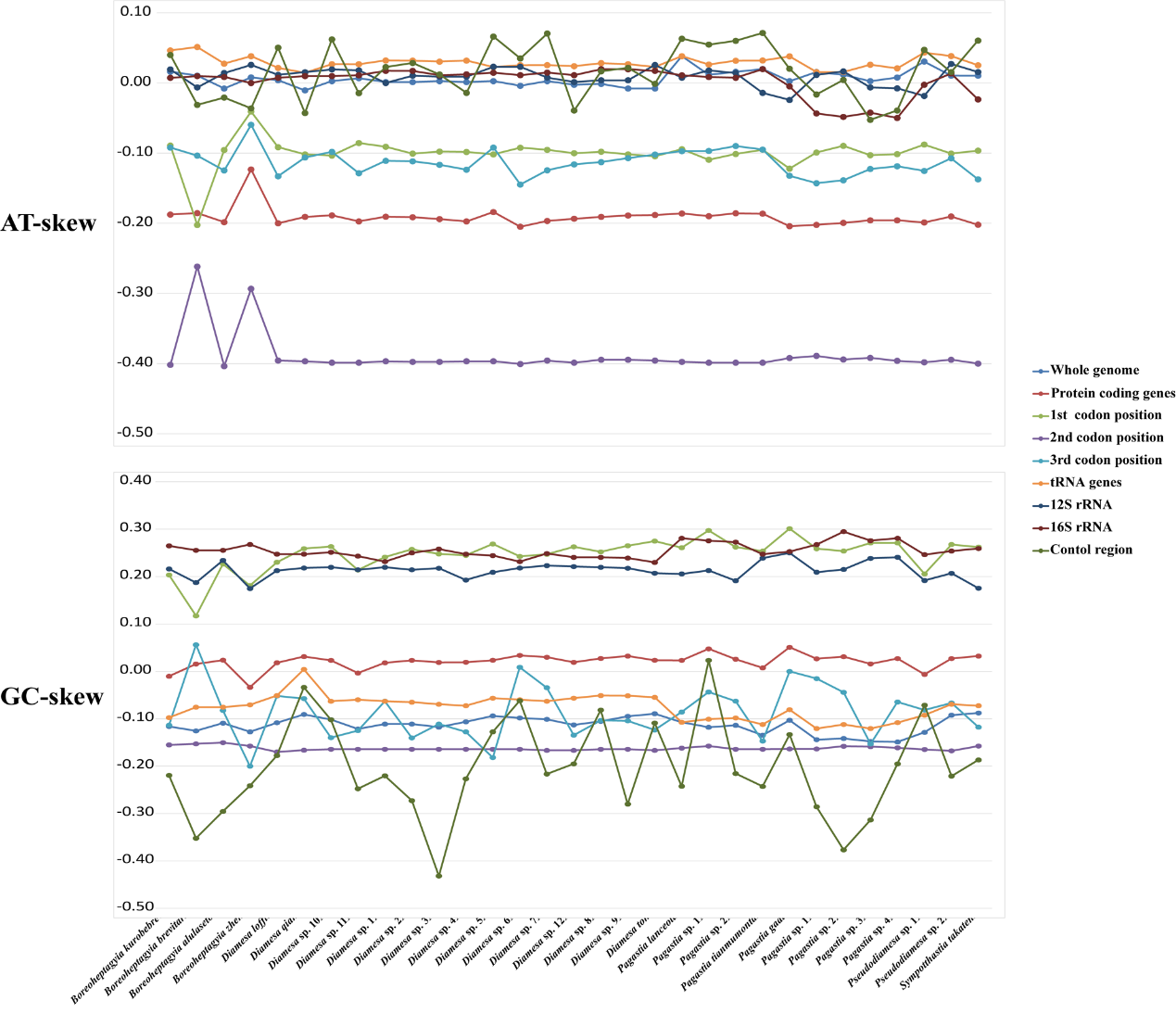


**Figure S2.** AT-skew and GC-skew of mitochondrial genes of Diamesinae species.


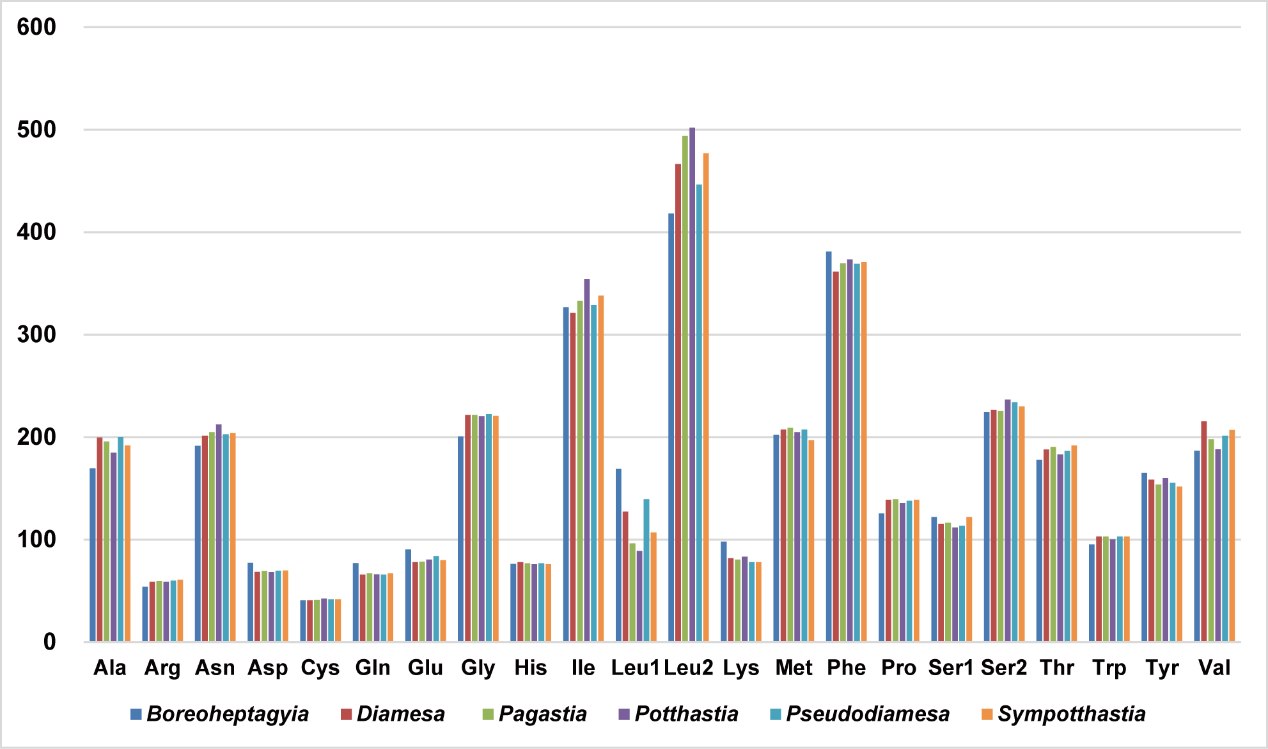


**Figure S3.** Patterns of codon usage of the Diamesinae mitogenomes. The X-axis shows the codon families and the Y-axis shows the total codons.
